# Human immunodeficiency virus and AIDS and other important predictors of maternal mortality in Mulago Hospital Complex Kampala Uganda

**DOI:** 10.1186/1471-2458-11-565

**Published:** 2011-07-14

**Authors:** Julius N Wandabwa, Pat Doyle, Benjamin Longo-Mbenza, Paul Kiondo, Betty Khainza, Emmanuel Othieno, Noreen Maconichie

**Affiliations:** 1 Department of Obstetrics and Gynaecology, Walter Sisulu University Private Bag X1 Mathatha 5117, South Africa; 2 Department of Epidemiology and population health, London School of Hygiene and Tropical Medicine, Keppel Street WC1E 7HT, UK; 3 Walter Sisulu University, Private Bag X1 Mthatha, 5117, South Africa; 4 Department of Obstetrics and Gynaecology, Makerere Medical School P.O.Box 7072, Kampala, Uganda; 5 Department of Anaesthesia, Makerere Medical School, P.O.Box 7072, Kampala, Uganda; 6 Department of Pathology, Makerere Medical School P.O.Box 7072, Kampala, Uganda; 7 Department of Epidemiology and population health, London School of Hygiene and Tropical Medicine, Keppel Street WC1E 7HT, UK

## Abstract

**Background:**

Women with severe maternal morbidity are at high risk of dying. Quality and prompt management and sometimes luck have been suggested to reduce on the risk of dying. The objective of the study was to identify the direct and indirect causes of severe maternal morbidity, predictors of progression from severe maternal morbidity to maternal mortality in Mulago hospital, Kampala, Uganda.

**Methods:**

This was a longitudinal follow up study at the Mulago hospital's Department of Obstetrics and Gynaecology. Participants were 499 with severe maternal morbidity admitted in Mulago hospital between 15^th ^November 2001 and 30^th ^November 2002 were identified, recruited and followed up until discharge or death. Potential prognostic factors were HIV status and CD4 cell counts, socio demographic characteristics, medical and gynaecological history, past and present obstetric history and intra- partum and postnatal care.

**Results:**

Severe pre eclampsia/eclampsia, obstructed labour and ruptured uterus, severe post partum haemorrhage, severe abruptio and placenta praevia, puerperal sepsis, post abortal sepsis and severe anaemia were the causes for the hospitalization of 499 mothers. The mortality incidence rate was 8% (n = 39), maternal mortality ratio of 7815/100,000 live births and the ratio of severe maternal morbidity to mortality was 12.8:1.

The independent predictors of maternal mortality were HIV/AIDS (OR 5.1 95% CI 2-12.8), non attendance of antenatal care (OR 4.0, 95% CI 1.3-9.2), non use of oxytocics (OR 4.0, 95% CI 1.7-9.7), lack of essential drugs (OR 3.6, 95% CI 1.1-11.3) and non availability of blood for transfusion (OR 53.7, 95% CI (15.7-183.9) and delivery of amale baby (OR 4.0, 95% CI 1.6-10.1).

**Conclusion:**

The predictors of progression from severe maternal morbidity to mortalitywere: residing far from hospital, low socio economic status, non attendance of antenatal care, poor intrapartum care, and HIV/AIDS.

There is need to improve on the referral system, economic empowerment of women and to offer comprehensive emergency obstetric care so as to reduce the maternal morbidity and mortality in our community.

## Background

Severe maternal morbidity also known as near miss is defined as a very ill pregnant or recently delivered woman who would have died had it not been that good care or luck was on her side [1-4].

Analysis of maternal deaths has long been used for evaluation of women's health and obstetric care. Investigation of severe maternal morbidity can also be used to review the performance of a health facility with regard to the management and implementation of medical protocols or interventions put in place to prevent maternal mortality [3,5,6]. Every year an estimated 514,000 women die from pregnancy related causes and 80-99% of these occur in developing countries [7], and an estimated twenty million women suffer acute obstetric morbidity. The majority of maternal deaths world-wide are attributed to direct obstetric causes, such as haemorrhage, obstructed labour, eclampsia, unsafe abortion, sepsis and medical conditions especially in this era of HIV/AIDS [7-11]. Maternal mortality has been attributed to low socio economic class, low educational status, lack of antenatal care and lack of women empowerment [10-13]. Factors that put a woman with severe maternal morbidity to the risk of mortality have been attributed to delay of patients to arrive in hospital and delay to receive treatment [14]. Delay in receiving care is really crucial to the survival of the woman with severe maternal morbidity [15,16]. The quality of care factors in hospitals which predispose mothers with severe morbidity to progress to mortality include poor institutional deficiencies such as shortage of supplies, equipment, staffing and some are administrative. These have been reported to be responsible for most maternal deaths in Sri Lanka, contributing to 66% of maternal deaths [17], and similar findings have been reported in South Africa [18] and Tanzania [11]. In the UK, a study was done to investigate quality of care factors before admission to the intensive care unit. Sub-optimal care was identified as a cause of severe morbidity. The care problems identified were failure of organisation, lack of knowledge by health workers, failure to appreciate clinical urgency, lack of supervision and failure to seek advice [15]. Severe maternal morbidity is common in Ugandan hospitals and in some cases, mothers will progress to maternal mortality. Shortage of blood for transfusion has been a major problem in hospitals in Uganda since the advent of HIV/AIDS. Patients who need massive blood transfusion are at risk of not only death but possibility of HIV infection if blood transfused is in the window period of HIV. There is well established significant contribution of HIV/AIDS to maternal mortality in developing countries including Uganda but the predicting impact of HIV/AIDS on maternal mortality is not well documented. It is important therefore to study the predictors for progression of severe maternal morbidity to mortality so as to prevent maternal mortality in our community.

## Methods

### Design

This was a longitudinal follow up study of cases of severe maternal morbidity admitted from 15th November 2001 to 30th November 2002.

### Setting

Mulago Hospital labour wards. Mulago Hospital is a National Referral Hospital for Uganda and a teaching Hospital for Makerere University College of Health Sciences. It is also a district hospital for Kampala City Council. Women who develop complications in and around Kampala city are referred here for management.

### Study population

Were women who had come to deliver at Mulago Hospital. Participants in this study were mothers with severe morbidity admitted to the study. Severe maternal morbidity were defined as pregnancy related life threatening conditions such as severe pre-eclampsia/eclampsia, severe obstructed labour, ruptured uterus, severe postpartum haemorrhage, and severe abruption placenta, placenta preavia with severe vaginal bleeding, severe anaemia and puerperal sepsis.

### Selection of the participants

The cases were selected daily from the labour suite and the emergency gynaecological wards as long as they fulfilled the selection criteria of individual causes of severe maternal morbidity described somewhere else[19]. The cases were followed up till discharge or death. They were interviewed about their socio demographic characteristics, social and family history, gynaecological and surgical operations, medical conditions, past and present obstetric performance. Information on the quality of care was obtained including antenatal attendance and investigations done, referral status, use of a partograph, cadre of the delivery attendant, shortages of drugs, and blood, the waiting time for operation and the reasons for the delay and the presence of a senior doctor. For those who were too sick, their spouses or first relatives were interviewed and later when the patients improved they were interviewed on discharge. The waiting time for, duration of operation were recorded by the study midwife.

Follow-up of the cases: All cases were followed up by the study midwives daily and they obtained information on the progress and the management until the mother was discharged or died. Post mortem assessment was performed on those patients who died to confirm the cause of death. On discharge or death the clinical record files of the patients were reviewed and information on management were extracted. All the information from the interviews and record extraction was filled into a pre coded questionnaire. Maternal mortality was considered as the dependant outcome variable to be explained by independent predictors.

### Statistical analysis

Continuous variables were presented as means and standard deviations (SD) while categorical variables were presented as proportions (%). Student t- test was used to compare means. Chi square test was used to test the distribution of categorical variables and relative risks were calculated to identify univariate potential predictors of maternal mortality. Potential predictors of maternal mortality were placed in logistic regression model to exclude confounding factors and identify independent predictors of maternal mortality.

Age was included in these models so as to be consistent with other studies. All factors that were significant in the socio demographic characteristics were picked and placed in a model together with age and these were adjusted against all other factors in the study. The independent predictors for progression to maternal mortality were adjusted for distance from home to Mulago hospital, asking for permission to attend health clinic or hospital, patient's job, age of the patient, type of care and medical diseases.

The multivariate odds ratios (OR) were calculated with their 95% confidence intervals (95% CI) to demonstrate significant association between maternal mortality and identified independent predictors. Time to death, Cox regression and Kaplan Heist curves were not considered because of short follow up period. P values < 0.05 were regarded as significant. The data was analysed using STATA soft wear vesion 8 (STATA Inc, Tx, USA).

### Ethical Considerations

The study was approved by the research and ethics committees of Makerere University Faculty of Medicine, Mulago Hospital and National council of Science and Technology in Uganda. The participants gave written informed consent to participate in the study, whereby none were to be denied any healthcare in case they declined to participate or withdrew from the study.

## Results

The study followed up 499 mothers with severe morbidity. Of these 460 mothers survived while thirty nine died. This gave a ratio of severe maternal morbidity to maternal mortality of 12.8 to 1, incidence rate of 8% and maternal mortality ratio 7815/100,000.

The deaths were due to obstetric haemorrhage (30.9%), eclampsia (12.8%), obstructed labour and ruptured uterus (10.3%), puerperal sepsis (12.8%), anaemia (15.4%), anaesthetic complications (2.6%), and medical diseases (15.2%). The majority (69.4%) of deaths were as a result of direct obstetric causes. The majority of women in both groups were below 30 years of age; about 77% of women who died were between the ages of 20-29 years. The mean age of survivors was 22.3 (SD = 2.3 years) and those who died was 23.6 (SD = 4.7 years). There was no statistical difference in age between the two groups (P = 0.16).

There was no difference in the marital status, (P = 0.16), the husband's job (P = 0.45,) the education level of women (P = 0.52), type of house (Brick/cement versus mud P = 0.45). history of chronic hypertension (yes versus no, P = 0.36).

Use of contraception (yes versus no, P = 0.10). Past delivery of still birth (yes versus no, P = 0.15), Previous delivery by caesarean section (yes versus no, P = 0.28), hypertension in pregnancy (yes versus no, P = 0.10). Present obstetric performance where parity (low versus high, P = 0.07); referral to hospital (Mulago versus other centres, P = 0.60); birth weight (low versus normal, P = 0.17); level of delivery attendant (P = 0.32) were not significantly associated with maternal mortality (results not shown). However long distance (> 5 kilometres) from home to Mulago hospital, long distance (> 5 kilometres) to the nearest primary health unit, peasant mothers, seeking permission to visit health unit, family history of hypertension, short birth spacing, non attendance of antenatal care, not knowing how to respond to vaginal bleeding during pregnancy, not given oxytocics, male sex of baby, shortage of blood or its products, shortage of drugs including antibiotics and magnesium sulphate and presence of HIV/AIDS were identified as significant univariate potential predictors of maternal mortality. (Table 1) After adjusting for different confounding factors, short birth spacing less than 36 months, no response to vaginal bleeding during pregnancy, male sex baby, shortage of blood or its products, shortage of drugs including magnesium sulphate and antibiotics and presence of HIV/AIDS were significantly and independently associated with maternal mortality. (Table 2)

**Table 1:** Relative Risk (Rr) of Martenal Mortality Conferred by Univariate Predictors

VARIABLES IDENTIFIED	CRUDE ODDS RATIO (95% CI)	P VALUE
Long distance from home to Mulago (km)		
≤ 5 km	1.0 (-)	
> 5 km	2.9 (1.2-7.0)	0.03
Long distance to nearest health centre (km)		
≤ 5 km	1.0 (-)	
> 5 km	3.4 (1.2-10.0)	< 0.0001
Peasant mothers	3.4 (1.2-100)	< 0.0001
Working mothers	1.0 (-)	
Need to request permission to visit health unit/hospital		
Yes	2.4 (1.2-4.5)	< 0.0001
No	1.0 (-)	
Family Hypertension	1.0 (-)	
No		
Yes	2.5 (1.1-5)	0.02
Birth spacing		
≥ 36 months	1.0 (-)	< 0.0001
< 36 months	3.3 (1.1-10)	
Not attended Antenatal Care		
Yes	1.0 (-)	
No	5.9 (3.0-11.9)	< 0.0001
Don't know response to vaginal bleeding during pregnancy		
No	2.9 (1.2-7.0)	
Yes	1.0 (-)	0.02
Previous admission to hospital		
No	1.0 (-)	
Yes	2.6 (1.1-5.9)	0.03
Male Sex of baby		
Female	1.0 (-)	
Male	2.9 (1.3-6.3)	< 0.0001
Given oxytocics:		
Yes	1.0 (-)	
No	5.2 (2.3-11.6)	< 0.0001
Shortage of blood or its products	13.9	
Yes	13.9 (7.9-33.6)	< 0.0001
No	1.0 (-)	
Shortage of drugs, antibiotic, magnesium sulphate	7.1	
Yes	7.1 (3.0-17.1)	< 0.0001
No	1.0 (-)	
HIV Status		
Negative	1.0 (-)	
Positive	4.6 (2.2-9.3)	< 0.0001

**Table 2:** Independent Predictors of Maternal Mortality Identified by Multivariate Analysis

VARIABLES	CRUDE ODDS RATIO (95% CI)	P VALUE
Birth spacing		
≥ 36 months	2.5 (1.1-10.0)	< 0.0001
< 36 months	1.0 (-)	
Not attended Antenatal Care		
Yes	1.0 (-)	< 0.0001
No	4.0 (1.3-9.2)	
Don't know response to vaginal bleeding during pregnancy		
No	4.3 (1.2-7.0)	< 0.0001
Yes	1.0 (-)	
Sex of baby Female	1.0 (-)	
Male	4.0 (1.6-10.1)	< 0.0001
Given oxytocics:		
Yes	1.0 (-)	
No	4.0 (1.7-9.7)	< 0.0001
Shortage of blood or its products		
Yes	53.7 (15.7-183.9)	< 0.0001
No	1.0 (-)	
Shortage of drugs, antibiotic, magnesium sulphate		
Yes	3.6 (1.1-11.3)	< 0.0001
No	1.0 (-)	
HIV Status		
Negative	1.0 (-)	
Positive	5.1 (2.0-12.8)	< 0.0001

## Discussion

The Uganda safe mother hood initiative was introduced in the early 1990s with the aim of halving maternal mortality by the year 2000. This objective was not achieved since maternal mortality in Uganda has remained high. The most frequent cause of death was post- partum haemorrhage. This is similar to the finding in other developing countries [8,10,11,18]. Majority of fatal cases (85%) and non fatal (90%) were below the age of thirty years with a similar mean age. Severe maternal morbidity and maternal deaths afflict young women and this is similar to other reports from Sub Saharan Africa [10,12].

The further away the woman lived from Mulago hospital, the higher the risk of death with those living more than ten kilometres having a risk of three times to die. This is because they developed severe maternal morbidity conditions and when referred but arrived in a very critical condition and sometimes it was difficult to salvage them. Other studies have also reported that the further the distance the mother stayed from hospital the higher the risk of death [18,20]. A distance of 10 to 15 kilometres was not too far from a referral hospital, but due to lack of an effective referral system, women developed complications in a health unit and when referred, arrived late in hospital due to lack of ambulance system.

The delay in reaching the hospital has been attributed to women seeking permission from their spouses or their in-laws before they could access the health facility.

The women who sought permission before attending a health unit were associated with three times the risk of dying compared to those who didn't. Similar findings were reported from the study in Tanzania [12]. It is likely that seeking for permission before accessing a health facility at the time complications occurred may be a manifestation of lack of empowerment of the women as they could no make their own decisions.

Women who had some form of employment were associated with less risk of dying compared to the unemployed. The employed women had better education, were more likely to attend antenatal clinic and may be empowered and therefore carried less risks for maternal mortality. Low educational status, poverty and lack of empowerment have been reported to be associated with maternal mortality, [8,12,20-24].

The women who were admitted to hospital during the present pregnancy for any medical conditions were four times more likely to die compared to those who were not admitted. These women could have had medical conditions like malaria, hypertension or anaemia which predispose to mortality.

Birth interval of greater or equal to thirty six  months was associated with a reduced risk of dying relative to those whose birth interval was thirty six or less months. The reasons for this may be that prolonged birth interval promotes recovery of a woman from a previous pregnancy. This confirms that spacing most likely due to use of family planning was associated with reduction of maternal deaths.

Non-attendance of antenatal care was found to be associated with an increased risk of maternal death compared to those who had attended antenatal care. Antenatal care is essential to screen women for risk factors for obstetric complications and those that do not benefit from this have an increased risk of maternal death. For antenatal care to be effective the patients should book in the first trimester and attend regularly as prescribed by the health provider.

When women were asked what they would do when they noticed vaginal bleeding during pregnancy, those who said that they would go to hospital were more likely to survive. Those who did not know what to do and where to go were associated with four times risk of dying compared to those who knew what to do. Bleeding during pregnancy is called ante- partum haemorrhage which needs to be evaluated and managed because of the un predictable outcome. Having complication during pregnancy has been reported as a predictor of maternal death [9].

The women who delivered male babies were associated with four times risk of dying compared to those who delivered female babies. It could be that male babies were bigger were associated with obstructed labour or ruptured uterus. In addition male babies were associated with pre- eclampsia and abruptio placenta and these complications predisposed the mothers to death [8-10,12,20].

Patients who did not get oxytocics soon after delivery were associated with four times risk of progressing to death compared to those who had it. The reason for this is that some patients delivered outside Mulago hospital and did not get oxytocics soon after delivery and presented with severe post partum haemorrhage. The other possible reason is that because of poor record keeping by staff and recall bias of patients could have caused some bias and this could not be a true reflection of the practice. However, the management of the third stage of labour if not accompanied by use of oxytocics is associated with increased chance of developing post- partum haemorrhage which is a major cause of maternal mortality.

The patients who needed blood but blood was not available for transfusion were associated with an increased risk of fifty four times of progression to maternal death compared to those who didn't require blood transfusion. Shortage of blood has been reported as one of the factors that have increased the maternal mortality in Mulago hospital [20]. The shortage has been made worse by the HIV prevalence in Uganda as blood donated have a big proportion of high HIV positivity. Shortage of blood has also been reported to increase maternal deaths in Nakuru provisional general hospital in Kenya [25]. Review of other studies show shortage of blood as a major cause of maternal deaths [26].

Puerperal sepsis contributed to a bout 6% of the maternal deaths in this study. This is lower than that reported from Nigeria [10]. Puerperal sepsis can be reduced by having skilled delivery attendants and by carrying out delivery in a clean and aseptic environment. Patients with puerperal sepsis who needed specific antibiotics for their condition and were not available were associated with four times the risk of progressing to maternal mortality compared to those who received antibiotics in the hospital. This accounts for high proportion of deaths among mothers with puerperal sepsis. The shortages increased the patients delay in access of the right treatment which also predisposed them to maternal death. The most affected group of patients were those of the low income group who couldn't afford the cost of treatment.

The study found that HIV positive women were associate d with five times the risk of dying compared to those who were HIV negative. These results are similar to what has been reported in Uganda on the contribution of HIV to maternal mortality with odds ratio of 3.2 [27] and 5.4 [28]. This is similar to the results of Meta analysis of studies from developing countries [29]. However, this is in contrast to the study from Ireland which reported no effect of HIV on severe maternal morbidity cases that progressed to maternal death [30]. It is possible that patients who are HIV positive in Ireland are on antiretroviral drugs and their immunity is high and are not likely to experience severe maternal morbidity and mortality.

### Limitation and strength of study

The number of cases that died were few and this limited the study in some degree. However the predictors identified and recommendation made can be generalised in all tertiary hospitals but was not substantial enough to make a policy reform in prevention of maternal mortality.

## Conclusion

The predictors of progression from severe maternal morbidity to mortality were: residing far from hospital, low socio economic status, non attendance of antenatal care, poor intrapartum care, and HIV/AIDS.

There is need to improve on the economic empowerment of women, have an effective referral system in place, offer comprehensive obstetric care and strengthen prevention and management of HIV/AIDS so as to reduce on the severe maternal morbidity which may and progress to mortality in our country.

## Competing interests

The authors declare that they have no competing interests.

## Authors' contributions

JNW was PhD student who with PD and NM as supervisors conceptualized and designed the study; carried out the data collection and follow up of the study participants, the data analysis and revised the draft manuscripts. PK, BK and EO contributed to data collection, data analysis and interpretation and revision of the manuscripts. BL was involved in the in the data analysis and interpretation, drafting of the initial manuscript and subsequent revision of the manuscripts. All co-authors approved the final manuscript.

## Pre-publication history

The pre-publication history for this paper can be accessed here:

http://www.biomedcentral.com/1471-2458/11/565/prepub
